# Antibody–drug conjugates in colorectal cancer: molecular design, preclinical advances, and translational challenges

**DOI:** 10.1007/s00280-026-04906-9

**Published:** 2026-05-28

**Authors:** Md Arif Ansari, Md Abubakar, Mohd Mazharuddin Ansari, Nabeeha Koya, Janmejay Gupta, Maria Aazam, Sonali Sandeep Shinde, Sana Ahmed, Amita Rai, Krishna Murti, Biplab Pal, Sachchida Nand Rai, Palwinder Kaur, Smita Shenoy, Nitesh Kumar

**Affiliations:** 1 Department of Pharmacology, Kasturba Medical College, Manipal Academy of Higher Education, Manipal, India; 2 Department of Pharmacology and Toxicology, National Institute of Pharmaceutical Education and Research, Hajipur, Vaishali, Bihar, 844102 India; 3 Department of Pharmaceutical Chemistry, Dr. D. Y. Patil Institute of Pharmaceutical Sciences and Research, Pimpri, Pune, 411008 Maharashtra India; 4 Department of Pharmaceutics, Deccan School of Pharmacy, Darussalam, Aghapura, Nampally, Hyderabad, 500 001 Telangana India; 5 Department of Pharmaceutical Analysis, National Institute of Pharmaceutical Education and Research, Hajipur, Vaishali, Bihar, 844102 India; 6 Department of Pharmacy Practice, National Institute of Pharmaceutical Education and Research, Hajipur, Vaishali, Bihar, 844102 India; 7 Department of Pharmacy Practice, School of Pharmaceutical Science, Lovely Professional University, Phagwara, 144401 Punjab India; 8 University Centre for Research and Development (UCRD), Chandigarh University, Mohali, 140413 Punjab India; 9 School of Pharmaceutical Sciences, Lovely Professional University, Phagwara, 144411 Punjab India

**Keywords:** Antibody‒drug conjugates, Monoclonal antibodies, Colorectal cancer, EGFR

## Abstract

Colorectal cancer (CRC) remains a leading cause of cancer-related morbidity and mortality worldwide, largely because of tumor heterogeneity, therapeutic resistance, and the limited efficacy of existing targeted therapies in advanced disease. Antibody–drug conjugates (ADCs) have emerged as a promising class of targeted anticancer therapeutics that combine the specificity of monoclonal antibodies with the cytotoxic potency of highly active payloads, thereby enabling selective tumor cell killing while minimizing systemic toxicity. Although ADCs have demonstrated substantial clinical success in several solid and hematological malignancies, their translation to CRC has been relatively limited, underscoring the need for a focused evaluation of CRC-specific ADC development. This review provides a comprehensive and critical overview of recent advances in ADC design and preclinical development for CRC, with emphasis on molecular architecture, linker–payload chemistry, antigen selection, and pharmacological mechanisms of action. Key components of ADCs, including antibody formats, cleavable and noncleavable linkers, and cytotoxic payload classes such as auristatins, maytansinoids, duocarmycins, and topoisomerase inhibitors, are discussed in the context of optimizing the therapeutic index and tumor selectivity. Mechanistic aspects of ADC activity, including antigen-mediated internalization, intracellular trafficking, payload release, and the bystander effect, are highlighted for their relevance to heterogeneous CRC tumors. This review systematically summarizes emerging preclinical ADC candidates targeting CRC-associated antigens, including EGFR, CEACAM5, c-Met, RON, DDR1, GPR56, LGR5, Claudin-2, and CD98hc. Finally, key translational challenges—including antigen heterogeneity, limited internalization, and off-target toxicity—are discussed alongside future perspectives emphasizing biomarker-driven patient selection and rational ADC design.

**Graphical abstract:**

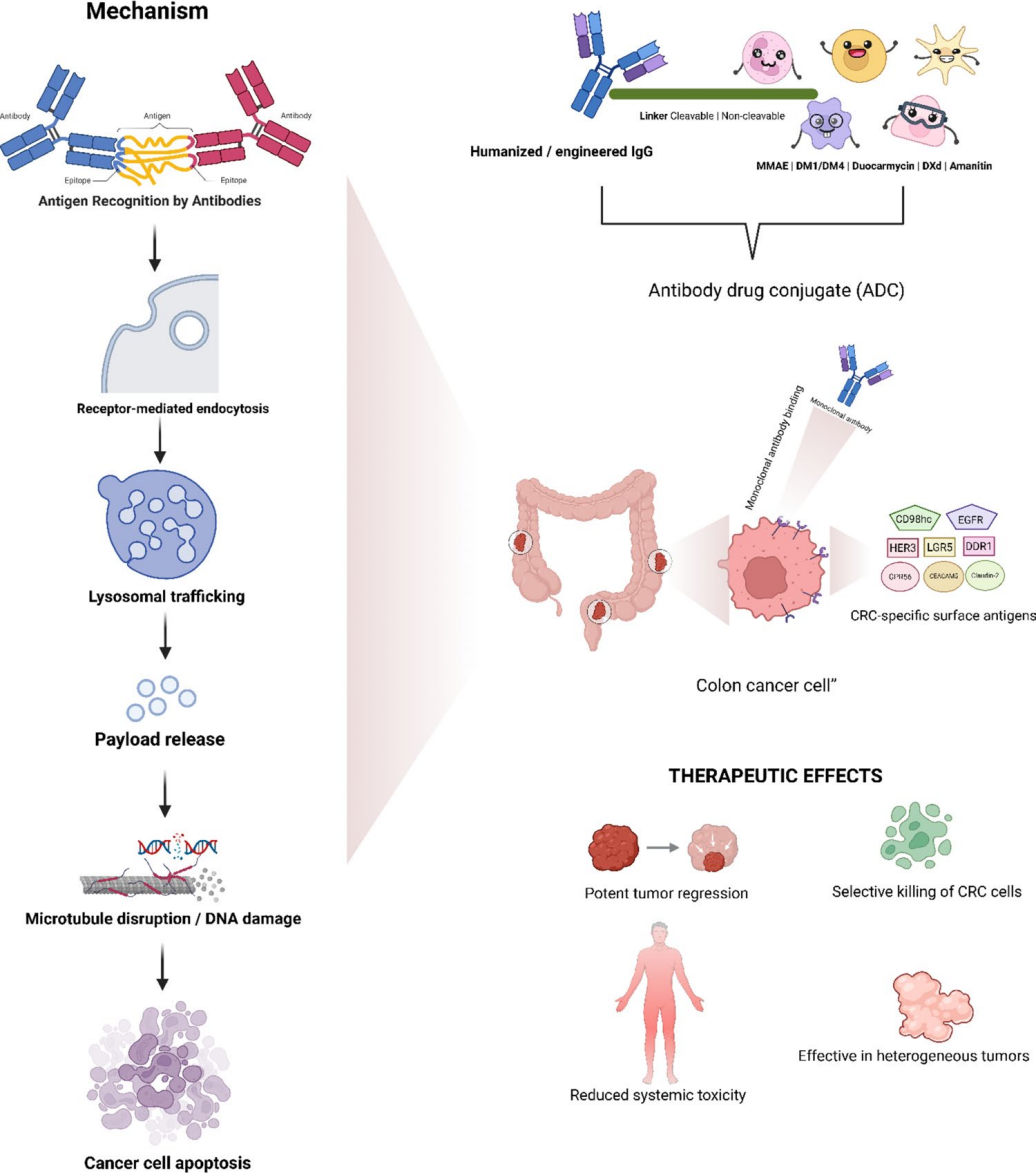

## Introduction

Globally, cancer remains the second most common cause of death, with millions of lives lost each year [1]. The global data for the year 2022 suggest that there were approximately 20 million new cases of cancer in addition to 10 million cancer-related deaths. Demographic predictions reveal that by 2050, there will be 35 million new cancer cases annually, a 77% increase compared with that in 2022 [2]. The United States (US) is anticipated to witness 2,041,910 new instances of cancer and 618,120 cancer-related fatalities in 2025. Among all cancer types, colorectal cancer (CRC) is the second most lethal and the third most common cancer worldwide. Globally, approximately 880,000 CRC-related deaths occurred in 2019, and 1.8 million new cases of this disease were reported. In terms of the rate of screening recovery, recent modeling research has projected that by 2040, there will be between 4,000 and 7,000 more colorectal cancer (CRC) deaths in the US. The epidemiological patterns of CRC may have altered because of the rapid aging of the world population and the increasing incidence of risk factors such as obesity, smoking, sedentary lifestyles, and especially unhealthy eating behaviors [3, 4].

Chemotherapy is still widely used for cancer treatment, but it faces obstacles such as nonspecific toxicity, drug resistance, and a limited therapeutic window [5, 6]. Since each form of cancer or tumor is different, determining the best course of action for each patient is challenging; however, it is necessary to achieve successful results. The sheer amount of newly authorized antibodies and innovative antibodies being tested for in the clinic serves as a stark reminder of this scenario. The concept of a “magic bullet” was proposed by the German scientist Paul Ehrlich in the early 1900s, even though anticancer medications are typically not selectively absorbed by tumor lesions. However, it took more than eight decades to bring this futuristic concept to reality [7–10]. Therefore, creating drug delivery systems that are highly efficient and have low systemic toxicity in the treatment of malignancies may be valuable for addressing this issue [11].

The effective development of cytotoxic compounds, which first appeared in the 1940s, and monoclonal antibodies (mAbs) during the 1970s laid the groundwork for this development. As a result, a variety of immunoconjugates have emerged, which are classified according to their specific effectors as immunotoxins, radioimmunoconjugates, immunocytokines, or “antibody–drug conjugates (ADCs).” Modern ADCs represent highly selective drug delivery systems [12]. These ADCs are involved in the delivery of toxic chemicals to tumor cells that express specific cancer antigens (Ag), thereby presumably increasing the effectiveness of chemotherapy and reducing systemic exposure and toxicity. Therefore, they are frequently characterized as targeted cytotoxic delivery vehicles. An ADC comprises three primary structural components: a linker, a cytotoxic agent (also known as the warhead or payload), and an antibody (Ab) [13–15].

Since their first approval, Gemtuzumab ozogamicin, in 2000 by the U.S. FDA, a substantial number of ADCs have been generated and marketed; as of now, 15 ADCs that have received FDA approval are accessible globally for solid tumors as well as hematological malignancies until 2024 (FDA-Approved Antibody-Drug Conjugates up to 2024 - BOC Sciences). Nevertheless, more than 100 ADCs are currently being evaluated in various clinical trials. According to economic research, the worldwide market for ADCs is expanding quickly. Approximately $7.82 billion in 2022, with an annual compound growth rate of 11.2% predicted between 2023 and 2030 [16, 17].

In this review, we begin by introducing the fundamental molecular structure of an ADC and discuss how the conjugation chemistry, intracellular trafficking of the payload, metabolism, safety profiles, and interactions among the linker, payload, and Abs influence the physicochemical characteristics of the final product. Afterwards, as next-generation cancer treatments, we highlight a few examples of innovative ADC designs, such as bispecific and dual-payload ADCs, which are now in early preclinical development targeting CRCs. These findings will inform researchers of the essential design concepts for creating safe and effective ADCs.

## Components and general mechanism of antibody–drug conjugates

### Components of ADCs

Monoclonal antibodies (mAbs) or Ab fragments are chemically linked to active molecules, such as cytotoxic drugs, using labile (cleavable) or nonlabile (noncleavable) linkers, creating powerful ADCs. These ADCs serve as highly effective antitumor agents, delivering chemotherapy drugs directly and selectively to cancer cells under the precise guidance of Abs with exceptional specificity and affinity [7, 18].

#### mAbs

The selection of Abs with reasonable Ag specificity is essential in ADC design. Cross-reactions between low-specificity antibodies and other Ag might result in off-target toxicity or quick clearance before they reach tumors [18]. Earlier, ADCs faced immune challenges because of mouse antibodies, prompting the development of humanized antibodies that combine human sequences with mouse Ag-binding regions. Despite these advances, immune reactions can still arise, prompting ongoing research into fully human or engineered antibodies. Emerging formats such as bispecific antibodies (BiTEs) also require careful evaluation for immunogenicity [19]. Among the five classes of immunotherapy (IgA, IgM, IgE, IgG, and IgD), IgG is preferred in ADCs because of its low immunogenicity, stability, and abundance. Its Fc region promotes medication stability and tailored delivery to cancerous cells [20, 21]. IgA, which is essential for mucosal immunity, can target related malignancies [18, 22]. IgM can be used to identify low-Ag-expressing tumor cells, but its size and complexity pose obstacles to drug conjugation and internalization [23]. However, a recent study produced IgM-ADC by specifically conjugating designed “selenocysteines” at the C-terminus of each heavy chain to treat chronic lymphocytic leukemia [24, 25]. IgD, with its short half-life, is under investigation for ADC use, but further research is needed [26]. IgE is also being investigated for cancer treatment; however, its allergic potential necessitates efforts to minimize hypersensitivity while maintaining antitumor efficacy [27, 28].

#### Linkers

Linkers are important for ADCs because they connect the mAb to the cytotoxic payload. This helps drugs stay stable, work better, and be released at the right place. They fall into two general classes: cleavable and noncleavable [29, 30]. Table 1 includes a few examples of linker conjugation bonds and the release mechanisms of some ADCs. Cleavable linkers reduce off-target toxicity by enabling drug release under tumour-specific conditions (acidic pH, protease activity, or reducing agents) [31]. Cleavable linkers are further subdivided into two classes: (i) chemically cleavable linkers, including acid-sensitive linkers (e.g., N-acyl hydrazone in gemtuzumab ozogamicin) that are hydrolyzed in lysosomes [30, 32]; disulfide linkers that respond to intracellular glutathione [33]; and linkers that are cleaved by exogenous triggers (such as palladium and tetrazine) to control drug release [34, 35]. Rossin et al. [34] used pegylated tetrazine to achieve 90% drug release within 20 h, which was superior to that of enzyme-cleavable ADCs in mice. These developments demonstrate the possibility of external triggers for reliable and successful ADC therapies. (ii) Enzyme-cleavable linkers include dipeptide linkers (e.g., Val-Cit in Adcetris) that use tumor proteases [36, 37]; β-glucuronidase-cleavable linkers improve plasma stability and reduce aggregation, even at high drug-to-antibody ratios (DARs), more than traditional dipeptide linkers do [38]; β-galactosidase linkers target specific tumor types by overexpressing the β-galactosidase enzyme [39]; and phosphatase-cleavable linkers release hydrophilic payloads [40].

Noncleavable linkers, on the other hand (mostly aliphatic and polymeric), depend on the complete breakdown of the Ab within the cell for drug release, which is less efficient and can result in incomplete release, thereby reducing overall effectiveness. The lack of a bystander effect in tumors with unequal Ag expression may also restrict the effectiveness of noncleavable linkers despite their increased bloodstream stability and decreased off-target toxicity [11, 15, 41]. Types include methyl ester linkers (forms stable ester bonds) [42]; maleimide linkers (form thioether bonds via Michael addition with thiols) [43]; amide linkers (ensures drug retention until lysosomal degradation) [44]; amino acid linkers (engineered for enzymatic or pH-sensitive cleavage) [45]; and hydrophilic linkers (improving solubility and pharmacokinetics) [46].

**Table 1:** A few examples of linker conjugation bond types, including the release mechanism of some ADCs

Linker type	Conjugation bond	Release mechanism	ADC molecules	References
Cleavable (acid-labile)	Hydrazone	Cleaved in acidic conditions	Gemtuzumab ozogamicin	[47]
Cleavable (reduction-sensitive)	Disulfide	Cleaved by a reducing mechanism (glutathione)	Inotuzumab ozogamicin	[48]
Cleavable (protease-sensitive)	Peptide	Cleaved by protease (cathepsin B)	Brentuximab vedotin	[49]
Noncleavable	Thioether	Released after lysosomal degradation	Trastuzumab emtansine	[50]
Noncleavable	Amide	Release after complete ADC degradation	Trastuzumab emtansine	[50]

#### Payload

The payload is a key part of an ADC, and it must be highly potent and practical, with IC50 values ranging from 0.1 to 1 nM. They also must remain stable in the body and have functional groups that can easily attach to the Ab [15, 51]. Modern ADC payloads are classified into four main types: auristatins and maytansinoids, which target microtubules; calicheamicins, which induce DNA cleavage; and camptothecins, which inhibit topoisomerase-1 [11]. Newer auristatin derivatives, such as “monomethyl auristatin E (MMAE)” and “monomethyl auristatin F (MMAF)”, improve stability and efficacy, whereas advancements in maytansinoids, such as hydrophilic linkers and derivatives such as maytansine (DM1) and DM4, help transcend challenges such as drug resistance [52]. Other microtubule-targeting agents, such as tubulysins, cryptophycins, and hemiasterlin, disrupt cell division. DNA-damaging payloads, such as “calicheamicins” and “duocarmycins,” are highly potent and kill cancer cells through DNA breaks, alkylation, and cross-linking, even at lower Ag levels [53]. These emerging payloads, along with dual payload techniques and immune-stimulating agents, enhance ADC efficacy, offering more effective and safer cancer therapies.

### General mechanism of ADCs: Targeting, internalization, and release

**Fig. 1:**
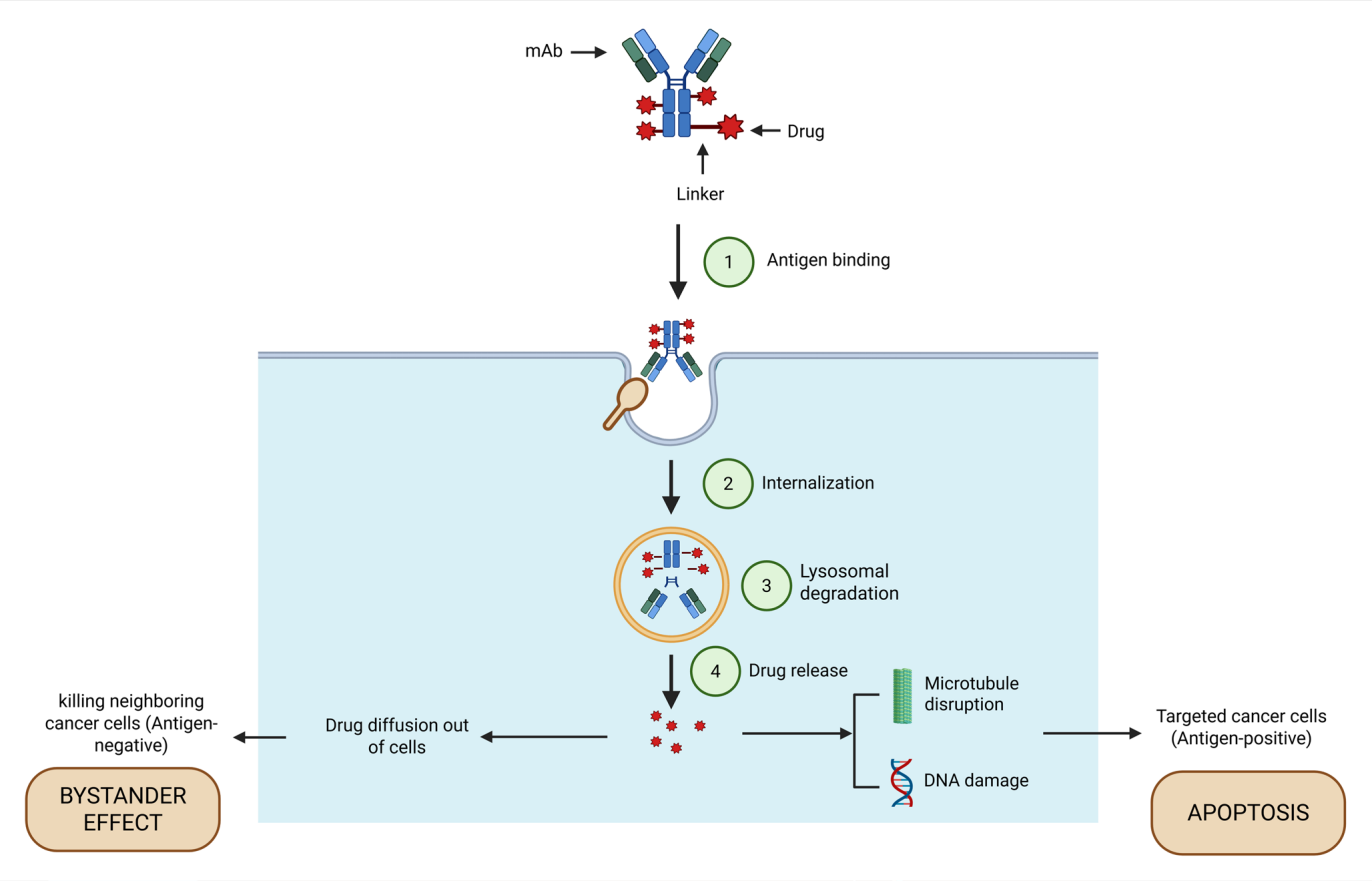
ADCs operate through a general mechanism. The binding of mAbs to the drug molecule at a specific binding site triggers internalization. The drug is released during lysosomal degradation, leading to nucleic acid damage and microtubule disruption, ultimately causing apoptosis in cancerous cells. The release of drugs also causes the necrosis of nearby cells

ADCs are intended to minimize harm to healthy tissues while delivering potent cytotoxic drugs directly to malignant cells. ADCs circulate after administration and bind to specific Ag on the surface of cancer cells. The “ADC–Ag complex” that results from this binding is taken up by endocytosis in three steps: (1) bud development; (2) membrane folding and vesicle maturation; and (3) scission and release into the cytoplasm [54]. Two main endocytosis pathways are involved in ADC internalization: “clathrin-mediated endocytosis (CME)” and “clathrin-independent endocytosis” [54, 55]. Once within the tumor cell, the ADC is broken down in the lysosome, delivering a cytotoxic substance that may cause oxidative stress, interfere with microtubule activity, and promote DNA replication, ultimately leading to apoptosis [56]. ADCs can also address epigenetic heterogeneity by increasing their efficacy against tumor cells that surround target Ag-expressing cells via the bystander effect, with cleavable junctions and hydrophobic payloads being critical for the creation of new ADCs [57, 58] (Fig. 1).

In a nutshell, for a practical ADC, the target Ag must be predominantly expressed on cancer cells and at sufficient levels (~ 10,000 copies per cell) to support binding and internalization. Ab should have excellent specificity and affinity while being nonimmunogenic. The cytotoxic payload must be extremely potent, since just 1–2% of it reaches the target within the cell. Optimizing the DAR and utilizing innovative cytotoxic compounds are critical to enhancing ADC effectiveness, making them attractive cancer therapeutics [59, 60].

## ADCs in pre-clinical studies

### Anti-EGFR ADCs

With the advancement of new treatments, epidermal growth factor receptor (EGFR) has become a prime target for a variety of cancers, notably CRC [61–63], non-small cell lung cancer (NSCLC) [64–66], and head and neck squamous cell carcinoma (HNSCC) [67, 68], which exhibit aberrant EGFR overexpression. Erythroblastic leukemia viral oncogene homolog (ERBB) ligands activate EGFR/ERBB1, leading to diverse tumor malignancies, including CRC. Epiregulin (EREG), a 46-amino acid EGFR ligand, is expressed at high levels in malignancies, where it promotes EGFR-mediated adhesion, angiogenesis, migration, and proliferation. Therefore, EGFR is an effective target for the treatment of CRC metastases because these pathways are critical to the survival of cancer cells [69–71].

The bifunctional polyethylene glycol (PEG6) linker, featuring both “maleimide” and “N-hydroxysuccinamide (NHS) functional groups (Mal-PEG6-DM1-NHS),” was utilized by Hartimath, El-Sayed, et al. in 2019 for the first time to synthesize nimotuzumab-PEG6-DM1 (*henceforth abbreviated by the term NPD*) ADCs. DM1 functions as a powerful antitumour drug by interfering with mitosis and inhibiting the growth of tumor cells [72, 73]. Nimotuzumab, a humanized monoclonal Ab (mAb), targets domain III of HER1 (EGFR1/ErbB-1), which inhibits the proliferation of tumor cells [74]. They produced highly soluble ADCs containing only monomeric Ab molecules via the PEGylated linker. Maleimide provides DM1 with a stable, noncleavable thioether linker, while NHS interacts with the primary amines of the Ab. An eightfold excess of the linker relative to the Ab has been used for nimotuzumab-PEG6-DM1-Low (NPD-L) ADCs, whereas a 16-fold excess has been used for nimotuzumab-PEG6-DM1-High (NPD-H) ADCs. The DARs of NPD-L and NPD-H were 3.5 and 7.3, respectively [75]. The most potent combination for DLD-1, MDA-MB-468, and HT-29 cells was NPD-H. Both NPD-L and NPD-H were tolerated favorably at a single dose of up to 50 mg/kg, as per toxicity studies. DLD-1 xenografts were successfully eradicated *in vivo* by their NPD-L and NPD-H conjugates. The combination of reduced EGFR affinity and decreased tumor uptake is probably the reason why there was no discernible benefit of the increased drug ratio NPD-H in the *in vivo* study [75].

Tikum, Nambisan, et al. (2022) developed a similar anti-EGFR ADC, nimotuzumab-PEG6-DM1 (NPD), as Hartimath, El-Sayed et al. [75]. Anti-EGFR monoclonal antibodies, such as matuzumab and nimotuzumab, interact with distinct epitopes within EGFR domain III. The conjugation procedures that produced DFO-matuzumab and NPD were conducted in 2 M HEPES (pH 8) and 2 M sodium carbonate (pH 9) buffers, respectively. The effectiveness of NPD in a DAR of 3–5 was shown in a prior investigation conducted by his group. *In vitro* cytotoxicity data revealed that, across all investigated cell lines except for HT-29 cells, the IC50 values of NPD were considerably lower than those of nimotuzumab. Three 15 mg/kg doses of NPD were given to mice harboring SNU-C2B xenografts. In two out of three mice, the treatment completely reversed the SNU-C2B tumor. Nimotuzumab and matuzumab do not compete with one another and can be taken together [76]. We were unable to locate any clinical studies about nimotuzumab-PEG6-DM1. However, we eventually found a related clinical trial evaluating trastuzumab-MCC-DM1 for CRC treatment, which is currently in phase II (NCT02465060).

Benelli, Costa, et al. (2022) created the Cet-ZA ADC. Zoledronate (ZA) coupled with cetuximab (Cet) was achieved by synthesizing a phosphoramidate that links a small molecule to an Ab and by using the chemical processes that occur in nucleic acids and proteins. Cet-ZA is comparable to second-generation ADCs because of its covalent linkage. The DAR was estimated at ~ 4.3. The data showed that the affinity of Cet for EGFR is unaffected by ZA conjugation and that the specificity of the original unconjugated Ab is preserved in Cet-ZA. Ultimately, in the HCT116 cell line, the internalization of both native Cet and Cet-ZA began at 4 h, and by 24 h, colocalization with the endosomal marker LAMP-1 was observed. This ADC increases the accuracy of malignant cell targeting by triggering an immunological response against the tumor that attracts effector cells to the tumor site [77]. When autologous or allogenic T lymphocytes and CRC organoids produce BTN3A1/2 molecules, coculture with Cet-ZA effectively stimulates γδ T-cell growth through the production of IPP and binding to Vδ2 TCRs. Remarkably, the Cet-ZA ADC triggered TCR-mediated cytotoxicity because of the effects of ZA and Ab-dependent cellular cytotoxicity (ADCC) because of the binding of Cet to FcγRIIIA/CD16, resulting in a dual effect on tumor cells [77, 78].

Pisheh, Matis, et al. (2024) covalently coupled aminobisphosphonates (N-BPs)—including zoledronate (ZA), risedronate (RIS), and ibandronate (IBA)—to cetuximab (Cet) without the use of a linker. Conjugation relies on interactions between the phosphoric acid groups of the N-BPs and the unbound amino groups on the Ab [79]. Hence, these ADCs do not involve any linker molecules. The average DARs for Cet-IBA, Cet-RIS, and Cet-ZA were 5.7, 7.1, and 3.5, respectively [79]. *In vitro* studies used two experimental methods to assess the direct effects of ADCs on the CRC cell lines Caco-2, DLD-1, and HCT-116. Cet al.one reduced Caco-2 cell proliferation by 50%, whereas Cet-N-BP ADCs enhanced this inhibition to nearly 90% after 120 h. However, neither the Ab alone nor the ADCs significantly inhibited the proliferation of SW-48, Colo-205, Colo-320DM, HCT-116, or DLD-1 cells. Researchers have also employed 3D spheroid cultures to better simulate in vivo conditions. Although Cet-ZA had the lowest DAR (3.5), it exhibited the greatest cytotoxicity toward CRC spheroids, followed by Cet-RIS and Cet-IBA. Notably, compared with the effects on the DLD-1 and HCT-116 cell lines, the cytotoxicity toward Caco-2 cells, which have a wild-type EGFR pathway, was less effective. The potential of ADCs to promote the growth of Vδ2 T cells generated from CRC tumors was also investigated in this work. Vδ2 T cell growth peaked at 25 days after being cultured with ADCs and IL-2. Compared with Cet-IBA and Cet-RIS, Cet-ZA enhanced T-cell proliferation [79]. Together, these results suggest that N-BPs may be processed by CRC cells, effectively activate Vδ2 T cells, and induce cytotoxicity in CRC organoids derived from patients [80].

Jacob, Anami, et al. (2025) developed three potential candidates (“VC-PABC-DuoDM, EGC-PABC-DuoDM, and EGC-PABQ-DuoDM gluc”) with a variety of linkers for the construction of an ideal EREG ADC. These CRC-H231-based ADCs include duocarmycins as a reliable payload. These compounds were manufactured using microbial transglutaminase (MTGase) with a site-specific chemoenzymatic method. Briefly, linker-duocarmycin DM (DuoDM) payloads—containing either the clinically validated “dipeptide valine–citrulline (VC)” or a novel tripeptide linker, “glutamic acid–glycine–citrulline (EGC),” known to improve the hydrophilic nature and stability of the ADC—were attached to a branched bis-azido linker. This linker was coupled to H231 via strain-promoted alkyne–azide cycloaddition. The resulting conjugates, “EGC-cDuoDM” and “novel EGC-qDuoDM”, with a glucuronide linker, were successfully synthesized and purified, achieving > 95% product purity. Crucially, in contrast to cDuoDM, qDuoDM-gluc can act more like a hydrophilic prodrug upon β-glucuronidation. The homogeneity of each ADC was validated by ESI‒MS analysis, with a consistent DAR of 4 [81]. Regardless of RAS mutations, EREG ADCs with tripeptide linkers were most effective against CRC cells that expressed EREG. Preclinical safety and efficacy investigations on “CRC cell lines” and “patient-derived xenograft (PDX) models” revealed that these EREG ADCs were well tolerated, abolished EGFR pathway activity, substantially slowed or regressed tumor formation, and prolonged survival. These findings suggest that EREG is a viable target for the development of ADCs to combat CRC and other cancers with elevated EREG expression. EREG ADCs could have potential for both “RAS mutant” and “WT patients,” enhancing current therapeutic options, even though the effectiveness of clinically authorized anti-EGFR mAbs is primarily limited by RAS mutational status [81].

### Tmab-VcMMAE-SMCC-DM1

Tmab-VcMMAE-SMCC-DM1, a dual-payload conjugate derived from an ADC, Tmab-VcMMAE, which combines two antimitotic drugs, DM1 and MMAE, with trastuzumab through distinct linkers [82]. To create an ADC (Tmab-VcMMAE), trastuzumab was first coupled with the antimitotic drug MMAE by a cleavable linker (Val-Cit). The ADC, Tmab-VcMMAE, was then attached to a second antimitotic medication, DM1, using a noncleavable linker, Succinimidyl-4-(N-maleimidomethyl) cyclohexane-1-carboxylate (SMCC), to generate a dual conjugate (Tmab-VcMMAE-SMCC-DM1). UV‒Vis analysis revealed a DAR of 5.25 [82]. Compared with trastuzumab alone, this dual-payload conjugate demonstrates enhanced cytotoxicity, potentially overcoming tumor recurrence and treatment resistance through a dual conjugation strategy [31, 83, 84]. Integration of the VcMMAE moiety into Tmab enhances the ability of the ADC to penetrate and localize within targeted tissues, while attachment of SMCC-DM1 and VcMMAE aims to induce complete cell death and apoptosis [82]. *In vitro* examination of Tmab-VcMMAE-SMCC-DM1 against “HER2 + breast cancer cell lines” and “CRC cell lines” revealed that it was cytotoxic, with SK-BR-3 cells showing greater sensitivity to MMAE than to DM1 [85]. DLD-1 cells were susceptible to both free medicines and ADC. Owing to increased HER2 expression, SK-BR-3 cells were more resistant despite their sensitivity. The proliferation rate of CRC cells was decreased by a dual conjugation method that targeted microtubule disruption, while this unique ADC conjugate also preserved the tumor penetration capacity [82].

These findings suggest that a combination of two microtubule-targeting payloads with different modes of action may be able to combat drug resistance and seed tumor recurrence, paving the way for next-generation dual-payload conjugate ADCs. We have not found any clinical research on Tmab-VcMMAE-SMCC-DM1 in any cancer. However, two related studies were found. In accordance with a phase I clinical investigation in Chinese participants with advanced solid tumors, the Sigvotatug vedotin ADC is designed to deliver MMAE to cells that express integrin beta-6 (NCT06549816). The effectiveness and safety of recombinant humanized anti-HER2 mAb-MMAE coupling substance (RC48-ADC) in neoadjuvant therapy for patients with breast cancer that exhibit HER2 expression are being assessed in a phase II clinical study (NCT05134519).

### Anti‑CD98hc‑DM1

According to Montero, Del Carmen, et al. (2023), colon cancer has an overexpression of CD98hc, which may be addressed therapeutically. Therefore, they employed a method that has been effective in triple-negative breast cancer to investigate the possible antitumoral effect of targeting CD98hc in CRC [86]. This approach is based on the production of an ADC that combines the potent antimicrotubular drug DM1 with an Ab (anti-CD98hcECTO) that recognizes the CD98 ectodomain. Five CRC cell lines, namely, HT29, HCT116, SW480, SW620, and LoVo, were treated with varying doses of anti-CD98hc-DM1 for 4 days to evaluate the antiproliferative effect of the drug. All five CRC cell lines demonstrated a dose-dependent reduction in MTT metabolism, with IC50 values ranging from 3.16 to 4.06 nM. However, none of the examined cell lines showed a significant decrease in number upon treatment with the naked anti-CD98hc Ab (henceforth abbreviated as anti-CD98hc), indicating that the Ab payload is primarily responsible for this reduction in cell expansion. Three tumoral patient-derived organoid (PDO) cultures were treated with approximately 10 nM anti-CD98hc-DM1 for 4 days to assess the antiproliferative effect of the drug. In every instance, anti-CD98hc-DM1 Ab administration reduced cell viability in a dose-dependent manner. Its antiproliferative effect on normal organoids has been shown to be less potent than that on tumorous ones [86].

The impact of this ADC was next examined in two *in vivo *colon cancer models: an HT29 cell xenograft in nude mice and a colon cancer PDX model. In the first model, it resulted in a significant decrease in tumor development. On the other hand, neither tumor development nor survival was impacted when the mice were treated with either free medication, DM1, or the naked anti-CD98hc Ab. In the second model, ADC treatment significantly decreased tumor size development and increased survival in mice harboring known BT6224 PDXs [86]. In a nutshell, taking into account these characteristics, a drug–Ab combination that identified CD98h was developed. In both *in vitro* (cell lines and PDOs) and *in vivo* (xenografted nude mice and PDXs) CRC models, this modified Ab demonstrated anticancer efficacy. According to the data presented here, CD98hc could represent a new ADC target that could be added to the therapeutic arsenal against CRC, provided that well-planned clinical trials are being conducted.

### Anti-RON antibody Zt/g4-DM1

The receptor tyrosine kinase receptor d’origine nantais (RON) protects against cellular stress, plays a role in epithelial carcinogenesis through immunological regulation within the tumor immune microenvironment, and activates many oncogenic pathways. Alternatively, in both human and animal tissue models, suppression of RON reverses tumor growth. This makes RON a targetable protein that is very desirable for tumor therapy [87]. The production and pharmacological effectiveness of a new “anti-RON Ab Zt/g4–DM1” conjugate targeting CRC were reported by Feng, Yao et al. in 2014.

Zt/g4 (IgG1a/κ) was attached to DM1 by a thioether bond, resulting in Zt/g4–DM1, which exhibited a DAR of 4:1. Zt/g4-DM1-induced RON endocytosis is required to transfer DM1 within CRC cells. *In vitro* studies revealed that in three CRC cell lines (HT29, SW620, and HCT116), Zt/g4–DM1 led to a gradual decrease in cell-surface RON over time. However, LoVo cells did not demonstrate specific binding. Cell viability studies revealed that the Zt/g4–DM1 IC50 values for HT29, HCT116, and SW620 cells were 1.64, 2.16, and 4.03 µg/mL, respectively, after 72 h. Zt/g4-DM1 did not affect RON-negative LoVo cells [88]. Single-dose treatment with 20 mg/kg ADC suppressed tumor growth by 82–95% and delayed tumor growth by approximately 2 weeks in mouse xenograft models using the HCT116, HT29, and SW620 cell lines. *In vivo*, it is estimated that 5 mg/kg Zt/g4-DM1 is required to prevent tumor development. Multiple dose-ranging experiments revealed that the Q4D × 5 regimen at 7 mg/kg (35 mg/kg in total) significantly inhibited tumor development. A better therapeutic index was achieved by increasing the dosage to 10 and 15 mg/kg (total doses of 50 and 75 mg/kg, respectively). These outcomes yielded an IC₅₀ of 5.01 mg/kg, which is in line with the ~ 5 mg/kg estimate from single-dose experiments. These findings inform the formulation of optimal dosage regimens for the possible clinical advancement of humanized Zt/g4-DM1. A toxicity study revealed that it is well tolerated at therapeutic levels, with negligible behavioral or weight effects, probably due to limited conjugate dissociation because it is incapable of binding mouse RON. A single dosage of 60 mg/kg resulted in considerable weight loss (6–19%), indicating a maximum cumulative dose for safe repeated dosing. This helps determine clinical dosage limits [88].

### T_4_H_11_-DM4

T4H11-DM4 is an ADC that targets discoidin domain receptor 1 (DDR1), a tyrosine kinase that is more highly expressed in various cancer types, including CRCs. DDR1’s cell-surface distribution and rapid endocytosis make it a potential target Ag for ADCs, spurring research into DDR1-targeted ADCs for colon cancer treatment [89–91]. The very effective microtubule inhibitor DM4 was coupled to T4H11 via a cleavable disulfide linkage, “N-succinimidyl 4-(2-pyridylothio) butyrate (SPDB)”, to generate the anti-DDR1 ADC T4H11-DM4. The conventional lysine coupling approach was used to attach SPDB-DM4 to lysine residues accessible on the surface of T4H11. The mean DAR value was 3.3 mol·mol^− 1^ [89]. Flow cytometry (FCM) revealed that T4H11-DM4 interacts with tumor cells with the same selectivity as T4H11 does, and its internalization efficiency postbinding is equal to that of the unconjugated Ab, as established in the HT-29 and HCT116 cell lines. The findings revealed that T4H11-DM4 was highly cytotoxic to colon cancer cells *in vitro*. HT-29 cells were susceptible to this ADC, with an IC50 of 2.5 nM [89]. Mouse xenograft tumor models were created utilizing the DDR1-positive colon cancer cell lines HT-29, HCT116, and HCT15. The mice were given three doses of T4H11-DM4. Complete tumor regression was detected in the HT-29 and HCT116 cells at 5 and 10 mg/kg, with ~ 60% tumor volume inhibition at a minimum dosage of 2.5 mg/kg. There was no significant difference from the control IgG-DM4 (10 mg/kg). T4H11-DM4 inhibited the HCT15 tumor model in a dose-dependent manner (22%, 60%, and 90% at 2.5, 5, and 10 mg/kg, respectively). This ADC has also been tested against platinum-resistant CRCs. T4H11-DM4 efficiently inhibited the growth of platinum-resistant SW480-OR and HCT116-OR cells, with IC50 values of 56.9 ± 6.4 nM and 21.2 ± 12.1 nM, respectively. In SW480-OR xenografts, three doses of 5 or 10 mg/kg resulted in complete tumor shrinkage, whereas unconjugated mAb and oxaliplatin had a limited effect. These findings illustrate the ability of ADCs to deliver cytotoxic payloads to DDR1-expressing cells [89]. Preclinical safety investigations demonstrated that T4H11-DM4 seemed relatively safe at therapeutic levels in BALB/c nude mice. It was well tolerated at concentrations up to 50 mg.kg^− 1^, with negligible effects on animal behavior and body weight. A single dose of T4H11-DM4 at 70 mg.kg^− 1^ reduced the body weight of the BALB/c mice by approximately 10%. This dosage restriction may be a helpful guide for preclinical safety studies in non-rodent species [89].

In short, this ADC demonstrated significant anticancer activity both *in vitro* and *in vivo* while maintaining an optimal safety profile. It has also been shown to have anticancer activity against platinum-resistant CRCs. These results indicate that anti-DDR1 ADC is an appealing approach for the management of CRC.

### H6-DM4

H6-DM4 is a type of anti-5T4 ADC that was first studied by Wang, Lai et al. in 2018. The antimitotic cytotoxin, maytansinoid DM4, and a chimeric anti-5T4 mAb are connected to this ADC (H6-DM4) via a cleavable SPDB linker that is susceptible to an intracellular reducing environment and releases a lipophilic adduct, S-methyl-DM4, which has the potential for a bystander effect [92]. In summary, the synthesis involved coupling reactions by adding an excess of 7–10 times the molar mass of SPDB-DM4 to the Ab at a final concentration of 5 mg/mL in PBS containing EDTA and 10% DMF (v/v) and letting it sit at room temperature for the whole night, yielding H6-DM4, which had an average DAR of approximately 2.7 mol/mol [93]. In the *in vitro* cytotoxicity assay, H6-DM4 exhibited high potency in the nanomolar range against 5T4^+^ cancer cells, with IC50s of 0.53 nM for DLD-1 cells, 3.89 nM for HT-29 cells, and 1.74 nM for BXPC3 cells, among others. However, it demonstrated poor cytotoxicity toward 5T4-negative LoVo cells, with an IC50 exceeding 300 nM. To assess the anticancer efficacy of H6-DM4, GI cancer cells or PDXs were subcutaneously inoculated into nude mice, NOD-SCID, or NSG mice. H6-DM4 at 2.5 mg/kg or 10 mg/kg eliminated seven distinct GI cancer xenograft models. Almost all animals carrying GI cancer xenografts that expressed high levels of 5T4, such as PANC-1, BX-PC3, HCT-15, and DLD-1, were eliminated by H6-DM4 at a dose of 2.5 mg/kg. Furthermore, H6-DM4 treatment resulted in long-lasting tumor shrinkage that persisted for approximately 100 days after the end of treatment. For tumor cells that expressed lower or moderate amounts of 5T4, such as HGC-27, HT-29, and PDX-954, 10 mg/kg H6-DM4 resulted in total tumor regression [93]. There was no discernible morbidity or substantial weight loss throughout the course of therapy. The preclinical safety profile of H6-DM4 was evaluated at two doses: 2.5 mg/kg and 10 mg/kg. After euthanization on days 3 and 29 (for acute and delayed toxicity, respectively), there were no effects on the blood, liver, kidneys, marrow, or vital organs, indicating neither immediate nor delayed toxicity [93].

### U3-1402

U3-1402 ADCs, commonly referred to as patritumab deruxtecan, were first reported by Daiichi Sankyo, a Japanese pharmaceutical company [94]. The chemistry of U3-1402 involves an Ab component, patritumab (U3-1287), a humanized mAb targeting HER3, a protein on the cancer cell surface that is covalently coupled to a linker (MAAA-1162a). This drug linker, containing a tetrapeptide-based linker, joins the Ab to the cytotoxic payload, which includes a drug component (MAAA-1181a). The synthetic U3-1402 DAR was approximately 8. DXd, a derivative of the “topoisomerase I inhibitor, exatecan,” serves as the cytotoxic payload here [95, 96]. Initially, this ADC binds specifically to HER3, triggering a cascade of events that leads to its internalization into cancer cells via endocytosis. Remarkably, the internalization efficiency exceeded 50%, indicating a robust uptake mechanism. Within the intracellular milieu, ADC subsequently undergoes trafficking, culminating in its localization within lysosomes, which are responsible for degradation and recycling. Moreover, the payload of the ADC is released from the lysosome, which is facilitated by the acidic environment. This activation of DXd within the cancer cell interior ultimately hinders cancer cell growth [94, 97]. It has been shown to result in lasting tumor reduction and is well received by patients with HER3-positive breast cancer [NCT02980341]. These findings strongly support the exploration of the efficacy of U3-1402 in individuals with HER3-positive CRC. ADMET studies are ongoing to examine the pharmacokinetics and pharmacodynamics of this ADC. In xenograft models using athymic nude or SCID mice, U3-1402 is administered intravenously (IV) at a dosage of 5.6 mg/kg every 3 weeks [96]. In extensive twelve-week repeated-dose toxicity trials, both rats and cynomolgus monkeys were subjected to ADC administration. The majority of adverse effects are limited to GI and bone marrow toxicity, with a reversible trend occurring within a six-week post-treatment period. GI manifestations primarily include single-cell necrosis in GI crypts or the mucosal epithelium, whereas bone marrow toxicity is characterized by hematopoietic cell depletion and an elevated reticulocyte ratio. Notably, the fatality of a single-stage rat at 194 mg/kg resulted from severe liver injury and lymphatic/hematopoietic organ atrophy, whereas monkeys tolerated doses of up to 30 mg/kg without significant sequelae. Overall, U3-1402 evinced a commendable safety profile in these extensive chronic studies [94, 98].

The clinical study of U3-1402 was discontinued early because the preliminary evaluation for part 1 (signal finding) did not meet predetermined criteria; therefore, it will not proceed to part 2 (NCT04479436). However, clinical studies are currently underway for Unresectable Locally Advanced or Metastatic Breast Cancer (ICARUS BREAST) (NCT04965766), Operable Breast Cancer (NCT04610528), and Metastatic or Unresectable NSCLC (NCT03260491).

### GPR56

The GPR56 ADC is an innovative therapeutic designed to target CRC by exploiting the high expression of the GPR56 receptor in tumor cells [99]. This ADC comprises the mAb 10C7, which binds specifically to the extracellular domain of GPR56, and the DNA-damaging agent duocarmycin, which is linked via a protease-cleavable linker. This linker, composed of maleimidocaproyl (MC), Val-Cit, para-aminobenzyloxycarbonyl (PAB), dimethylethanolamine (DMEA), and PEG, is used to ensure that the cytotoxic cargo is selectively delivered into tumor cells. The DAR achieved was ~ 3.54. The ADC selectively binds to GPR56 on CRC cells and is internalized and releases the duocarmycin payload intracellularly, inducing DNA damage and cell death in a GPR56-dependent manner [99–103]. The ADC demonstrates potent cytotoxicity specifically against GPR56-expressing “CRC cell lines” and “patient-derived tumor organoids,” with minimal effects on GPR56-negative cells. Its antitumor efficacy has been validated in xenograft models, in which it significantly inhibited tumor growth (TGI) at doses ranging from 1.5 mg/kg to 5 mg/kg when administered weekly. Notably, at 2.5 mg/kg, the ADC achieved approximately 50% TGI in the SW403 model, and at 5 mg/kg, it induced slight tumor regression, followed by stasis, in the SW620 model [99–103]. However, some CRC cell lines, such as HCT15 and LS180, exhibit intrinsic resistance, potentially because of inherent resistance to duocarmycin. Furthermore, the efficacy of ADC depends on GPR56 expression levels in tumors, which are upregulated in 66–80% of CRC tumors, particularly in microsatellite-stable (MSS), non-CIMP, and chromosomal instability subtypes. This limits the application of the ADC to patients with high GPR56 expression. Preliminary safety studies in animal models suggest that the ADC is well tolerated, with no significant off-target effects observed. However, the lack of cross-reactivity with mouse GPR56 may lead to the underestimation of potential toxicity in humans, highlighting the need for thorough clinical evaluation. The potential for the development of drug resistance and the necessity for biomarker-driven patient selection underscore the challenges associated with clinical application expression [99, 103, 104]. Despite these challenges, this ADC has promising therapeutic potential for CRC patients, particularly those whose subtypes are associated with poor prognosis, warranting further clinical development and investigation. In our search on clinicaltrials.gov, we did not find any studies on this ADC.

### Anti-LGR5 MC vc PAB MMAE and anti-LGR5 NMS818

In 1998, Hsu et al. first discovered leucine-rich repeat G-protein-coupled receptor 5 (LGR5) [105]. LGR5 expression is increased in various cancerous tissues, including ovarian, colorectal, hepatocellular, and basal cell carcinomas [106–109]. It acts as a putative indicator of cancer stem cells in the early molecular stages of adenomatous lesions and the development of carcinogenesis in sporadic CRC [110]. Therefore, it can serve as a marker and enhance canonical Wnt signaling, thereby promoting cancer stem cell division and self-renewal. Thus, it can be targeted by ADCs. Gong et al. and Junttila and her collaborators used this gene as a therapeutic target. They developed anti-LGR5-specific ADCs that eradicated LGR5-positive tumors in various tissue types, including gastrointestinal (GI) cancer cell lines, and stopped recurrence in xenograft models of CRCs.

Gong et al. (2016) reported that two distinct ADCs, “anti-LGR5-mc-vc-PAB-MMAE” and “anti-LGR5-mp-MMAE,” were produced as a result of conjugating the anti-LGR5 mAb to MMAE using the cathepsin B protease-sensitive linker “maleimidocaproyl-valine-citrulline-p-aminobenzyloxycarbonyl (mc-vc-PAB)” and a noncleavable “maleimidopropionyl” linker, respectively. MMAE was effectively functionalized using amaleimidopropionyl linker for the latter conjugation approach, and under partial reduction conditions, it was subsequently coupled to free thiols of the anti-LGR5 mAb. LGR5-overexpressing cancer cells (293T-LGR4 and -hLGR5) exhibited cytotoxicity. Lysosomes rapidly take up both ADCs and bind LGR5 with nanomolar affinity in GI cancer cells. *In vitro* data suggest that cleavable “anti-LGR5-mc-vc-PAB-MMAE” was ten to twenty times more effective at destroying cancerous GI cells. The observed variation in potency may be attributed to the superior drug-release efficiency of “enzyme-mediated proteolytic cleavage,” such as that of lysosomal cathepsins, compared with the lysosome-mediated degradation of noncleavable ADCs [111]. Anti-LGR5-mc-vc-PAB-MMAE eradicated tumors and decreased their volume in a colon cancer “xenograft model” without harming healthy intestinal tissue or causing relapses. These investigations demonstrated that interconversion between LGR5^+^ and LGR5^−^ CSCs was responsible for tumor recurrence after ADC treatment [92, 112]. In summary, anti-LGR5-mc-vc-PAB-MMAE has demonstrated potential as a new therapy for targeting CSCs, eliminating LGR5^+^ tumors across multiple tissue types, and preventing relapse in GI cancer cell lines and a colon cancer xenograft model.

Junttila and her associates have also produced a specific anti-LGR5 Ab that has been coupled to two cleavable linker drugs with distinct mechanisms of action: the DNA-damaging anthracycline, PNU159682, and the antimitotic microtubule inhibitor, MMAE. Two anti-LGR5-specific ADCs were produced as a consequence, which are known as “anti-LGR5-MC-vc-PAB-MMAE (anti-LGR5-vc-MMAE)” and “anti-LGR5-NMS818” [113]. The first incorporated a humanized anti-LGR5 mAb conjugated to MMAE via the cysteines that typically form the mAb’s interchain disulfides, using a cleavable Val-Cit linker. As a result, this conjugate and the one discussed by Gong et al. (2016) are similar [111]. The same mAb is used in the second conjugation, NMS818, which is linked to the C-14 hydroxyl of the DNA-binding protein, PNU159682, by an acid-sensitive linkage and an engineered cysteine on the Ab heavy chain. The DARs of “MC-vc-PAB-MMAE” and “NMS818” ADCs were approximately 3.5 and 2, respectively [114, 115]. *In vitro* investigations have indicated that the free form of the medication produced from “anti-LGR5-NMS818” is significantly more effective against normal cells than it is against anti-LGR5-vc-MMAE, potentially affecting a greater number of cells [116]. Anti-LGR5-MMAE was found to be effective *in vivo* without impacting homeostatic epithelia or other tissues that express LGR5. Conversely, the “NMS818 ADC” demonstrated target-dependent toxicity and was in line with the established expression pattern of “LGR5”. The well-tolerated removal of intestinal LGR5^+^ cells may account for the absence of gut toxicity observed with anti-LGR5-MMAE. LGR5^+^ cells and bystander cells may have been eliminated together, accounting for the “NMS818” target-dependent toxicity in hepatic and GI tissues. Notably, because LGR5 depletion has been linked to liver and intestinal toxicity, these animals have not been viable for a long time [117, 118]. Upon internalization, both ADCs release membrane-permeable medication and may also exert a bystander effect on nearby cells. However, in regard to cell multiplication, notably normal LGR5 + cells, the free drug produced by NMS818 is 10–100 times more potent than MMAE. This might account for the higher toxicity of anti-NMS818 than of anti-LGR5-MMAE [119]. In conclusion, both xenografts and genetically modified mouse models of colon cancer demonstrated antitumour efficacy; the latter more closely resembled the situation observed in human tumors. Tumors with heterogeneous, low LGR5 expression levels also showed antitumour efficacy. Crucially, changes in tumor size did not occur immediately; instead, prolonged therapy was needed, indicating that reducing the number of CSCs takes longer than targeting non-CSCs does. No studies are currently available for any of the ADCs discussed here, even though clinical trials with a monotherapy arm have finished. However, owing to the sponsor’s decision, the combination arm was not implemented (NCT02726334).

### aPDL1–PLG–MMAE

New attempts to improve the therapeutic effectiveness of ADCs include the use of Ab-targeted nanoparticle delivery systems, which combine polymer nanocarriers with Abs to form antibody–nanogel conjugates (ANCs), as proposed [120]. Therefore, Zhang et al. (2023) introduced a nanomedicine, “aPDL1–PLG–MMAE,” that efficiently targets tumors with elevated PDL1 expression while delivering MMAE. Fc–PLG–MMAE was produced via amide condensation of poly(L-glutamic acid) (PLG) with Val–Cit–PAB–MMAE and the Fc–III–4 C peptide. The other two nanomedicines were developed by simply combining mAbs (aPDL1 with IgG) with Fc–PLG–MMAE in PBS for 4 h to produce antibody–PLG–MMAE nanomedicines (aPDL1–PLG–MMAE and IgG–PLG–MMAE), with DARs of approximately 13. *In vitro* studies on the cytotoxicity of PDL1-PLG-MMAE revealed that it was highly toxic to MC38 cells, with an IC50 of 12.14 nM. Because of its fast endocytosis and precise binding to the PDL1 protein present in MC38 cells, aPDL1–PLG–MMAE exhibited substantial cytotoxicity. However, owing to their sluggish rate of endocytosis, “Fc–PLG–MMAE” and “IgG–PLG–MMAE” did not trigger MC38 cell death, even at high concentrations (100 nM). These findings show that in MC38 cells with strong PDL1 expression, aPDL1–PLG–MMAE has a more potent cytotoxic *effect. An in vivo* MC38 colon cancer model revealed that aPDL1–PLG–MMAE-1 and aPDL1–PLG–MMAE-2 significantly suppressed tumor development after 21 days of therapy, with greater tumor growth inhibition (TGI) in the aPDL1–PLG–MMAE-2-treated group [121]. To summarize, aPDL1–PLG–MMAE, a tumor-active-targeting nanomedicine, does not affect the structure of the Fab segment in antibodies. Moreover, aPDL1–PLG–MMAE does not exhibit appreciable toxic side effects or an extended duration of internal circulation, has a strong tumor targeting capacity, and has an exceptional tumor suppression rate of 92.3%; moreover, it can quickly detect MC38. However, to date, we have not found any related clinical trials on this ADC.

### anti-GCC ATAC^®^s

This novel family of ADCs possesses amatoxin as the payload, known as ATAC^®^s (amatoxin-based ADCs). *Amanita phalloides*, the infamous “green death-cap” mushroom, contains the main toxin, α-amanitin, which selectively inhibits “eukaryotic RNA polymerase II (RNAP2)” [122]. “RNAP2” is necessary for both cellular development and homeostasis. α-Amanitin inhibits RNAP2, killing proliferating and quiescent cells by causing rapid proteolytic breakdown and, ultimately, cell death [123]. Using maleimide chemistry, an amatoxin derivative was conjugated to either a cleavable (ATAC^®^ I) or noncleavable linker (ATAC^®^ II), together with cysteine-engineered anti-GCC Abs, to create two GCC-targeting ADCs with amanitin-based payloads. Homogeneous ATAC^®^s with a DAR of 2.0 were produced using this site-specific conjugation technique. *In vitro* cytotoxicity results for both ADCs were positive, demonstrating picomolar activity against the GCC^+^ cells and no cytotoxicity against the GCC^−^ cells. In xenograft models at low doses, a single ATAC^®^ I dose resulted in temporally complete tumor remission. Following the administration of ATAC^®^ II at a dosage of 6 mg/kg, comparable antitumour efficacy was observed. A single dosage of “anti-GCC ATAC^®^ I (MTD: 7.5 mg/kg)” and “anti-GCC ATAC^®^ II (MTD: 50 mg/kg)” at ½ the MTD resulted in a slight remission of the tumor. The antitumor activity in both CRC PDX models was considerably enhanced by multiple doses of both ATAC^®^s at a lower dosage of ¼ MTD-delivered q7dx4. “Anti-GCC ATAC^®^s” are highly tolerable and have a high therapeutic index, as indicated by their safety profile in cynomolgus monkeys [124]. There are also no clinical trials related to this topic.

### Anti-Claudin-2 ADCs

Deregulation of the tight junction protein family, claudins (CLDN1–CLDN24), has been linked to tumorigenesis in several cancer types. It has been demonstrated that CLDN1 [125], CLDN2 [126], CLDN4 [127], and CLDN18 isoform 18.2 [128] can either be aberrantly expressed or elevated in CRC. Both CLDN1 and CLDN3-7 are expressed in the normal colon. While some claudins, such as CLDN6 and CLDN7, have been detected in CRC, their levels are lower than those in normal colons [128].

Gastric tumors and CRCs—two solid tumors that commonly spread to the liver—have high levels of Claudin-2 [129, 130]. Hence, one possible target for treating solid tumors that metastasize to the liver is claudin-2. Thus, Tabariès, Robert et al. (2024) synthesized anti-Claudin-2 ADCs. PNU was chosen as the principal drug compound for five of the top-performing anti-Claudin-2 leads (C2-NRC-2, C2-NRC-19, C2-NRC-20, C2-NRC-25, and C2-NRC-27) for the study. The selected Abs were subsequently coupled to PNU to increase their potency as ADCs. A DAR of approximately 2.0 was achieved. Exogenous Claudin-2-expressing SW403 CRC cells responded most effectively to anti-Claudin-2 ADCs. Compared with the other two CRC cell lines, which have endogenous Claudin-2 levels, the sensitivity of the HT-29 cells to anti-Claudin-2 ADCs was greater. For each tested Ab, comprehensive dose‒response curves and superior target specificity ratios were obtained. Among the five anti-Claudin-2 leads that were investigated, two were chosen for extensive conjugation: C2-NRC-2, which attaches to extracellular loop 1, and C2-NRC-20, which attaches to extracellular loop 2. Crucially, when known CRC cell lines and PDX models of CRC liver metastases were used, PNU-coupled anti-Claudin-2 ADCs inhibited the formation of replacement-type CRC liver metastases *in vivo*. This development of ADCs targeting Claudin-2 appears to be a viable therapeutic approach for the management of individuals with replacement-type liver metastases from CRC. Despite showing promise in several preclinical models, PNU-159,682-conjugated antibodies are not yet being used in any clinical cancer trials. This might be partly explained by the toxicity observed at PNU-conjugated ADC doses above 2 mg/kg [131]. The toxicity of anti-Claudin-2 ADCs was also assessed. Similar to control mice, C2-NRC-2-PNU mice lost weight more noticeably. With the exception of peripheral blood neutrophil percentage and lymphocyte proportions, hematologic analysis revealed no discernible variations. Claudin-2 ADC treatment decreased the liver metastatic burden by 6.37- and 2.54-fold, respectively [131]. In summary, anti-Claudin-2 ADCs demonstrate therapeutic effectiveness at low dosages without causing significant toxicity. The anti-Claudin-2 antibodies discussed here are noteworthy for their ability to specifically target Claudin-2 levels observed in PDXs from CRC hepatic metastases with replacement-type characteristics. Table 2 summarizes all ADCs in preclinical investigations, including their molecular structures, where applicable.

**Table 2:** Summary of ADCs in preclinical investigations

Sr.No.	ADCs	mAb	Payload	Linker	Chemical structure	DAR	Ref.
1.	Nimotuzumab-PEG6-DM1- (High & Low)	Nimotuzumab	Maytansine (DM1)	Polyethene glycol (PEG6)		High = 7.3, and low = 3.5, respectively	[75] [76]
2.	Cet-ZA	Cetuximab	Zoledronate (ZA)	NA		~ 4.3	[77]
3.	Cet-IBA, Cet-RIS, and Cet-ZA	Cetuximab	Ibandronate (IBA), Risedronate (RIS), and Zoledronate (ZA), respectively	NA		5.7, 7.1, and 3.5, respectively	[79]
4.	“VC-PABC-DuoDM,” “EGC-PABC-DuoDM,” and “EGC-PABQ-DuoDM gluc”	EREG	Duocarmycin	branched bis-azido, tripeptides (glutamic acid–glycine–citrulline; EGC), dipeptides (valine–citrulline; VC)		4	[81]
5.	Tmab-VcMMAE-SMCC-DM1	Trastuzumab	Dual payload: monomethyl auristatin E (MMAE), and Maytansine (DM1)	SMCC and Val-Cit, respectively		5.25	[82]
6.	Anti-CD98hc-DM1	anti-CD98hc^ECTO^	Maytansine (DM1)	NA		NA	[86]
7.	Anti-RON antibody Zt/g4-DM1	Zt/g4 (IgG1a/κ)	Maytansine (DM1)	thioether		4:1	[88]
8.	T4H11-DM4	Anti-DDR1	Maytansinoid (DM4)	N-succinimidyl 4-(2-pyridylothio)butyrate (SPDB)		3.3	[89]
9.	H6-DM4	anti-5T4	DM4	SPDB		2.7	[93]
10.	U3-1402	Patritumab (U3-1287)	DXd	drug-linker (MAAA-1162a).		8	[94]
11.	GPR56 ADC	10C7	Duocarmycin	“Protease linker (maleimidocaproyl (MC),” “valine-citrulline (Val-Cit), para-aminobenzyloxycarbonyl (PAB),” “dimethylethanolamine (DMEA),” and “polyethene glycol (PEG)”		~ 3.54	[99]
12.	“anti-LGR5-mc-vc-PAB-MMAE,” and “anti-LGR5-mp-MMAE”	anti-LGR5	MMAE	“cathepsin B protease-sensitive linker maleimidocaproyl-valine-citrulline-p-aminobenzyloxycarbonyl (mc-vc-PAB),” and “maleimidopropionyl linker”, respectively		NA	[111]
13.	“anti-LGR5-vc-MMAE,” and “anti-LGR5-NMS818”	anti-LGR5	MMAE and anthracycline PNU159682, respectively	valine-citrulline linker, and engineered cysteine, respectively		3.5 and 2, respectively	[113]
14.	aPDL1–PLG–MMAE	Anti-PDL1	MMAE	poly(L-glutamic acid) (PLG)		~ 13	[121]
15.	anti-GCC ATAC^®^s	anti-GCC	amanitin-based payloads	cleavable (The ATAC^®^ I) or noncleavable linker (the ATAC^®^ II)		2	[124]
16.	anti-Claudin-2 ADC	anti-Claudin-2	PNU-159,682	Valine-Citruline (VC), cleavable linker		~ 2	[131]

## Conclusion & future perspectives

The development of ADCs for CRC has evolved from simple molecular design to complex techniques to overcome the complex pathological challenges of the disease. A comprehensive synthesis of the preclinical candidates presented in this review indicates major differences in target suitability, internalization efficiency and the potential to address tumor heterogeneity.

Target suitability remains a primary difference among current CRC-targeted ADCs. EGFR is a well-established target because of its high expression in CRC metastases, but its therapeutic applicability is often limited by RAS mutation status. New developments, including EREG-targeted ADCs (e.g., VC-PABC-DuoDM and EGC-PABQ-DuoDM), show greater translational potential because they induce robust antitumor activity regardless of RAS mutation. In contrast, LGR5 and GPR56 are highly selective targets for cancer stem cell (CSC) populations and microsatellite-stable (MSS) CRC subtypes, respectively. However, the translational potential of GPR56-targeted ADCs is hindered by the lack of cross-reactivity with murine GPR56, which may lead to an underestimation of systemic toxicity in preclinical animals.

The effectiveness of internalization determines the efficacy of ADCs, as the cytotoxic payload needs to enter the intracellular environment to exert its effects. The HER3-directed ADC U3-1402 (patritumab deruxtecan) and the DDR1-directed T4H11-DM4 are highly internalized, with U3-1402 achieving efficiencies > 50%. Similarly, anti-CD98hc-DM1 and anti-RON (Zt/g4-DM1) conjugates use rapid Ag-mediated endocytosis to release their payloads. In contrast, targets such as 5T4 (targeted by H6-DM4) and GCC (targeted by ATAC^®^s) require highly potent payloads or optimized linker‒payload combinations to overcome differences in Ag density and internalization kinetics.

Tumor heterogeneity is likely the most prominent issue in CRC therapy. First-generation ADCs with noncleavable linkers, such as nimotuzumab-PEG6-DM1 (NPD), rely on the complete degradation of the Ab and are typically limited by the absence of bystander activity. To overcome this, current techniques include cleavable linkers and hydrophobic payloads to create a bystander effect, as observed in the case of anti-LGR5-mc-vc-PAB-MMAE and H6-DM4 conjugates that can eradicate neighboring Ag-negative cells. Furthermore, dual-payload methods, such as Tmab-VcMMAE-SMCC-DM1, utilize two different antimitotic mechanisms (MMAE and DM1) to lower the likelihood of tumor recurrence and resistance. The inclusion of α-Amanitin in anti-GCC ATAC^®^s has an additional benefit in that it inhibits RNA polymerase II in both proliferating and quiescent cells, enabling a novel mechanism for the clearance of dormant cancer cells that are generally resistant to traditional microtubule-inhibiting payloads.

Finally, the selection of nanocarriers, such as the aPDL1–PLG–MMAE nanogel conjugate, embodies a transition to increasing the DAR to ~ 13, substantially higher than the usual DAR of 3–4 in most candidates such as Cet-ZA or T4H11-DM4. Although this increases the effectiveness of the cytotoxicity, the problem in translation is balancing high payload delivery with tolerable off-target damage. Collectively, these comparative insights underscore that the next generation of CRC therapy will require a rational integration of target biology, linker stability, and payload potency tailored to the specific clinical subtype and metastatic profile of the patient. Therefore, future ADC approvals may benefit from an integrated strategy that incorporates meticulous target selection, along with adjustments to the Ab, linker, and payload components, tailored to specific cancer types. There are now 17 clinical trials (in the recruiting stage) for ADCs in CRCs available on clinicaltrials.gov, and an increasing number of drugs are entering clinical testing. We anticipate a significant increase in the number of authorized ADCs over the next few years, despite these compounds being more challenging to develop than bare antibodies.

## Data Availability

No datasets were generated or analysed during the current study.
